# Rapidly Evolving Giant Dermatofibroma

**DOI:** 10.1155/2010/620910

**Published:** 2010-03-09

**Authors:** K. J. Lang, S. Lidder, M. Hofer, C. Graham, A. Taylor

**Affiliations:** ^1^Department of General Surgery, Stoke Mandeville Hospital, Mandeville Road, Aylesbury, Bucks, HP21 8AL, UK; ^2^Department of Plastic Surgery, Stoke Mandeville Hospital, Mandeville Road, Aylesbury, Bucks, HP21 8AL, UK; ^3^Department of Histopathology, Stoke Mandeville Hospital, Mandeville Road, Aylesbury, Bucks, HP21 8AL, UK

## Abstract

Dermatofibroma, also known as “fibrous histiocytoma”, is a benign dermal or subcutaneous poorly circumscribed proliferation of spindle-shaped fibroblasts and macrophages in the dermis. Although it is commonly present as a brownish nodule the legs of females, it may also arise on the upper extremities, trunk, and rarely on the head. The exact pathogenesis is unclear. However, it is widely believed that the originating insult to the dermis is a folliculitis, an arthropod bite, or an unspecified initial inflammatory condition. Giant dermatofibromas of greater than 5 cm in diameter are rare, with only 22 cases reported in the literature. We present a case of a rapidly evolving pedunculated mass in the groin of a male patient. Histological examination confirmed this to be a giant dermatofibroma. Though this specimen cannot is not confirmed as such, the cellular subtype is sometimes present as a larger lesion with anecdotal reports of local recurrence and distant metastases. The clinical and radiological features which were somewhat suspicious of malignancy are considered in the context of the definitive pathological diagnosis of a benign lesion.

## 1. Introduction

Dermatofibroma is a common, usually benign, small papular (<5 mm) skin lesion often found on the lower limbs [1]. A number of clinicopathological variants have been described which include the cellular variant representing less than 5% of the cases, but more commonly encountered in larger lesions [2]. We report a case of an unusually large dermatofibroma with an apparently rapid phase of growth which led to a clinical suspicion of malignancy.

## 2. Case Report

A 61-year-old man presented to his general practitioner with a history of a rapidly evolving groin swelling. He recalled a pea-sized lesion in his right groin, which had remained static over the past 40 years. There was no relevant past medical history and the patient remembered no preceding episode of trauma or local irritation. 

**Figure 1:**
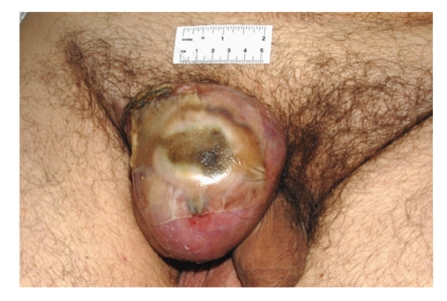
Anterior view of dermatofibroma (10.5 cm  ×  10 cm  ×  6 cm) in the right inguinal region. Ulcerated area is covered with tegapore dressing.

The lesion increased rapidly in size to a 10.5  ×  10  ×  6 cm firm, smooth exophytic mass with a shiny reddish surface. The centre was ulcerated discharging pus (Figure 1). The patient was referred for a surgical opinion. 

**Figure 2:**
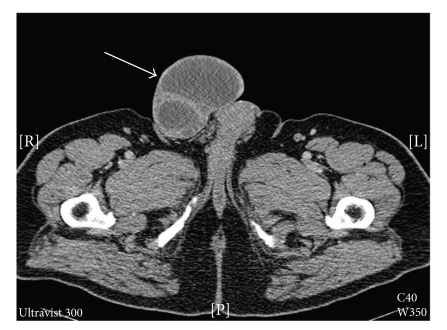
Axial computed-tomography view of dermatofibroma (arrowed) arising from the skin of the right base of the scrotum demonstrating a complex cystic structure with fluid and septations.

Computed tomography of the lesion showed a complex cystic structure with fluid and septations containing several foci of calcification (Figure 2). The lesion was excised under general anaesthetic. 

**Figure 3:**
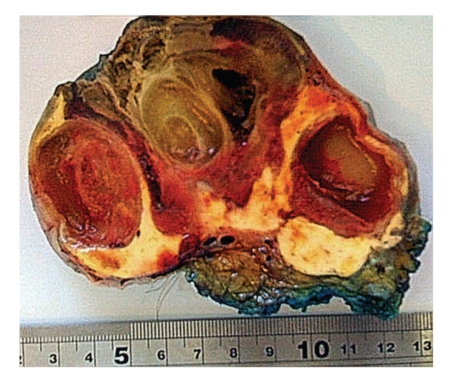
Excised tissue, sliced longitudinally, showing cystic and haemorrhagic areas.

Histological examination showed a macroscopically well-circumscribed, pedunculated, partly ulcerated lesion weighing 406.6 grams. On slicing, a well-circumscribed multilobulated mass with a variegated cut surface including cystic and haemorrhagic areas was apparent (Figure 3). Four tissue blocks were fixed for analysis.

**Figure 4:**
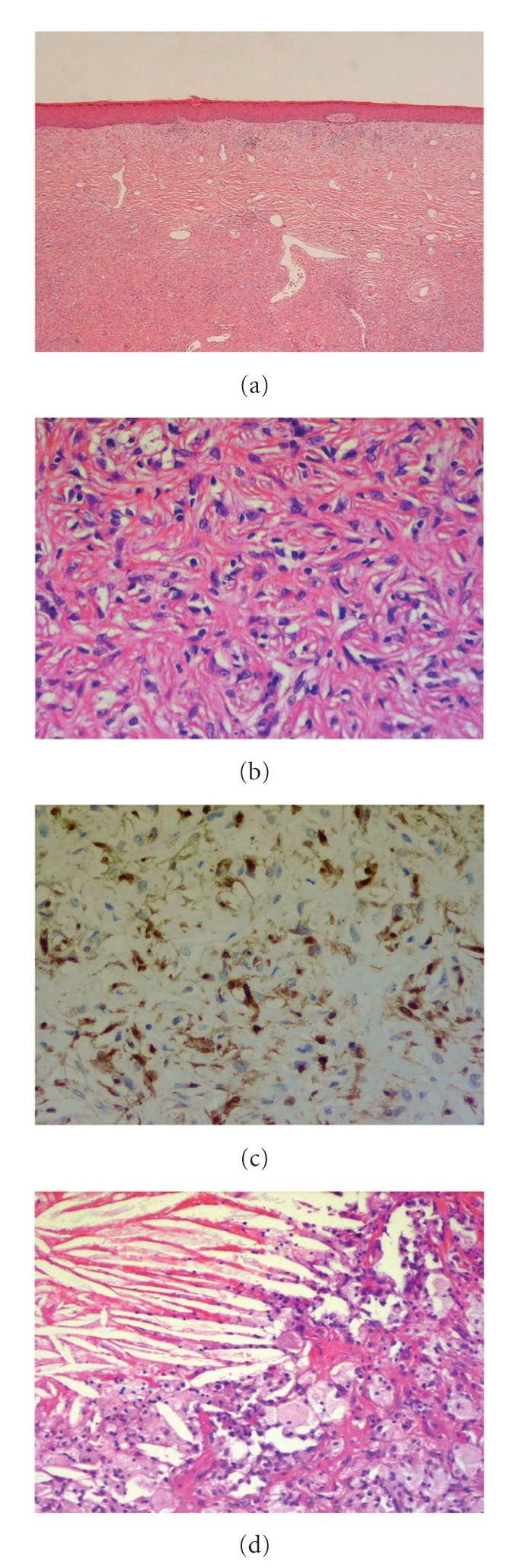
On low power the lesion is composed of spindled cells in the dermis with a well defined Grenz zone; H&E stain, moderate magnification ×40. On high power, bundles of interlacing spindle cells in the dermis. H&E stain, magnification ×100. Same area of lesion as in (b) stained for Factor XIIIa, showing diffuse staining with scattered strongly positive cells; Factor XIIIa stain, magnification ×100. High powered section of specimen showing cholesterol clefting and scattered giant cells; H&E stain, magnification ×100

All the tissue blocks were fixed in formalin and routinely processed for paraffin-embedding. Histological sections were stained with haematoxylin and eosin (H&E). Immunohistochemical studies were carried out using a standard panel of mesenchymal and melanocytic markers. Microscopically, the specimen contained a large spindle cell lesion extending from the dermis into the subcutaneous tissue. A well-defined Grenz zone was apparent away from the ulceration and bundles of interlacing spindle cells surrounding small groups of foamy macrophages were present. A storiform pattern was noted. At the centre of the lesion there were areas of cystic degeneration with focal haemorrhage, cholesterol clefting, and giant cells with focal calcification (Figure 4).

Immunohistochemical staining was positive for CD68 and factor XIIIa. The spindle cells stained strongly positive for factor XIIIa. Focal positivity with CD31 and SMA was noted. Staining for CD34, desmin, MNF116, S100 protein, and AE1/AE3 negative. The morphological and immunohistochemical features were those of a dermatofibroma showing marked degenerative changes. At three-month follow-up the patient was well with no evidence of recurrence.

## 3. Discussion

The most frequent clinical presentation of dermatofibroma is of a red-brown or yellow-brown papule on the leg measuring a few millimetres in diameter-which is mostly asymptomatic. Twenty percent of dermatofibromas occur in persons less than 17 years old. It almost invariably exhibits benign biologic behaviour. Clinically, it moves freely over the deeper layers on palpation and if the overlying epidermis is inwardly compressed, it exhibits the “dimple sign”, indicating tethering of the overlying epidermis to the underlying lesion. Pruritus and tenderness are not uncommon [2].

The aetiology of the lesion is controversial and was thought to represent a reactive process or dermal response to injury following an insult such as folliculitis, arthropod bites, or unspecified inflammatory conditions. However, recent cytogenetic studies demonstrating clonality suggest that these lesions may in fact be neoplastic [3]. Some prefer to think of dermatofibroma as a fibrosing dermatitis rather than a neoplasm [4, 5].

Histological findings in dermatofibroma typically include a degree of epidermal hyperplasia of the overlying epidermis. Sometimes, the basaloid proliferation simulates a basal cell carcinoma and the pathologist must be cautious of confusing these distinctly different entities. The dermis displays a poorly circumscribed proliferation of spindle cells associated with varying numbers of mononuclear cells. The line of differentiation of these spindle-shaped cells remains uncertain. These lesions are therefore classified as “fibrohistiocytic tumours” because of their appearance and the histopathological findings of a variable mixture of fibrocytes and macrophages. Foamy macrophages are commonly seen in varying numbers [1]. 

Cellular dermatofibroma is a rare subtype representing less than 5% of all fibrous histiocytomas. This pathological variant, while still favouring the lower limbs, is more common in males and most are less than 2 cm in diameter. When very large, the lesions may present clinically as a giant dermatofibroma. The features widely accepted as defining a giant dermatofibroma were first described by Requena et al [6] in their 1994 series of eight cases of dermatofibroma: (a) size >5 cm; (b) pedunculated; (c) benign biological behaviour despite its size; and (d) the same histopathological characteristics as conventional dermatofibroma. In the same series, none of the lesions excised recurred at an average of 35 months follow-up, suggesting that surgical management represents a satisfactory management strategy in giant dermatofibroma.

The differential diagnosis of dermatofibroma must include dermatofibrosarcoma protuberans. Immunohistochemical staining for CD34 is usually negative in benign lesions (positive in 85% of dermatofibrosarcoma protuberans) and may be the only distinguishing characteristic. However, it should be noted that cellular dermatofibroma may focally stain positive for CD34 though this is predominantly seen at the periphery of the tumour. Staining for factor XIIIa is positive in dermatofibroma and tends to be negative in dermatofibrosarcoma protuberans [7, 8]. Staining for smooth muscle actin is often focally positive in cellular dermatofibroma. By contrast, this stain is usually negative in cases of dermatofibrosarcoma protuberans.

We can speculate that an unrecognised inflammatory and/or haemorrhagic process in the period preceding presentation induced the rapid growth of the lesion in our patient. The speed of evolution of the lesion and the clinical findings did raise a suspicion of malignancy. However, preoperative computed tomography was more in keeping with a benign lesion. Because of the clinical concerns this lesion was excised with appropriate margins and submitted for pathological assessment. In light of the pathological diagnosis of a giant dermatofibroma (an aneurysmal fibrous histiocytoma) which appears excised no further treatment is indicated.
